# GLP-1 Receptor: A New Target for Sepsis

**DOI:** 10.3389/fphar.2021.706908

**Published:** 2021-07-14

**Authors:** Fuxun Yang, Fan Zeng, Xiaoxiu Luo, Yu Lei, Jiajia Li, Sen Lu, Xiaobo Huang, Yunping Lan, Rongan Liu

**Affiliations:** Department of ICU, Sichuan Provincial People’s Hospital, University of Electronic Science and Technology of China, Chengdu, China

**Keywords:** GLP-1 receptor, sepsis, hyperglycemia, GLP-1 receptor agonist, insulin

## Abstract

Patients with sepsis often exhibit hyperglycemia, which increases mortality. glucagon-like peptide-1 receptor agonists (GLP-1RAs) not only regulate blood glucose homeostasis but also improve organ dysfunction, regulate immunity, and control inflammation and other functions in patients with sepsis. Here, we review the possible application of GLP-1RAs in sepsis, to provide a new perspective for the clinical diagnosis and treatment of patients with sepsis complicated with stress hyperglycemia.

## Introduction

Sepsis is a dysfunctional response to infection that leads to life-threatening organ dysfunction (Seymour et al., 2016; Singer et al., 2016) with a mortality rate of more than 10%, of which 40% is due to septic shock (Seymour et al., 2016; Singer et al., 2016). Sepsis is often accompanied by stress hyperglycemia, which damages the immune response of the host, increases the risk of organ damage, and affects the prognosis of patients (Leonidou et al., 2007). A large number of studies on stress hyperglycemia (Sapolsky et al., 2000; Hunt and Ivy 2002; Jennifer et al., 2007; Saberi et al., 2008; Borst 2015; Rague. 2017) showed that stress hyperglycemia caused due to various reasons occurs through the whole pathophysiological process of sepsis, especially at the septic shock stage. Insulin is a commonly used drug to treat hyperglycemia, but excessive use of insulin could lead to frequent hypoglycemia and greatly increase the mortality of patients with sepsis (Ali et al., 2008). Therefore, the accurate regulation of the blood glucose level of the patients with sepsis is an urgent problem for clinicians.

Glucagon-like peptide-1 (GLP-1) receptor agonists (GLP-1RAs) are one of the new drugs used in the treatment of diabetes. The GLP-1 receptor is a member of the glucagon receptor family of G protein-coupled receptors (Mayo et al., 2003). The receptor protein consists of 463 amino acids. The receptor protein is glycosylated. Glycosylation can regulate the function of the receptor. GLP1R consists of two domains as follows: an extracellular domain, which binds to the C-terminal helix of GLP-1, and a transmembrane domain, which binds to the N-terminal region of GLP-1 (Mayo et al., 2003). Different domains in the third intracellular loop of the GLP-1 receptor are responsible for specific G protein coupling (Gαs, Gαi, and Gαo) (Hällbrink et al., 2001). In addition to the pancreatic tissue, the central nervous system, cardiovascular system, gastrointestinal tract, lung, kidney, thyroid, skin, and mesenchymal stem cells also express GLP-1 receptors (Wei and Mojsov 1995). GLP-1 receptors are activated by mimicking the endogenous GLP-1 to enhance insulin secretion and inhibit glucagon secretion in a glucose-dependent manner, as well as delay gastric emptying and reduce food intake through the suppression of appetite, thereby reducing blood sugar.

With recent studies, researchers have found that GLP-1RA has many functions in addition to regulating blood glucose homeostasis, such as improving organ dysfunction, regulating immunity, and controlling inflammatory response in patients with sepsis. However, the research and application of GLP-1RA in sepsis is limited, and the mechanism of action is still unclear. This paper intends to review its application in sepsis, to provide a new idea for the clinical diagnosis and treatment of patients with sepsis complicated with stress hyperglycemia.

## Mechanism of Action of GLP-1RAs

Enteroglycin is released primarily in response to enteral nutrition and promotes insulin secretion from pancreatic β cells in a glucose-dependent manner; for instance, through glucose-dependent insulin-stimulating peptide (GIP) and GLP-1. GIP is secreted by enteral endocrine cells (K cells), which are mainly located in the proximal small intestine and release GIP after enteral nutrition (including carbohydrates and fatty acids) (Campbell and Drucker 2013). GLP-1 is a member of a family of naturally occurring hormones, released after meals by the L cells in the small intestine (mainly composed of jejunum, ileum, and colon at the end of the lang Abraham’s cells secrete) and by the pancreas, which possesses a particular GLP-1 receptor, GLP-1R. GLP-1R stimulates adenylate cyclase and increases the level of cyclic AMP (cAMP) through Gαs (Drucker et al., 1987). It then stimulates protein kinase A (PKA)-dependent intracellular signaling, and cAMP-regulated guanine nucleotide exchange factor II (cAMP-GEFII, Epac2) directly activates the exchange protein. With the activation of the above signaling cascade, GLP-1 activates T cell nuclear factor. Insulin release and genetic changes are ultimately triggered through PKA- and calcineurin (CaN)-dependent pathways in pancreatic β cells (Lawrence et al., 2002; Holst 2007; Smith et al., 2019). GLP-1 inhibits ATP-regulated potassium channels through PKA and EPAC (cAMP-dependent mechanisms) (Holz 2004), leading to increased calcium influx, thereby increasing calcium-induced insulin secretion by stimulating glucose-dependent insulin release (Lee. 2014). In humans, GLP-1 has two bioactive forms, the 31-amino acid GLP-1 (7–37) and the 30-amino acid GLP-1 (7–36), with a difference of only one amino acid residue. Approximately 80% of the cyclic activity of GLP-1 comes from the GLP-1 (7–36) peptide (Talchai et al., 2012). The hypoglycemic effect of GLP-1 is glucose dependent. As an enterogenous hormone, GLP-1 can only exert its hypoglycemic effect when nutrients, especially carbohydrates, cause the blood glucose level to rise; however, it does not further reduce blood glucose when the blood glucose level is normal.

The new class of GLP-1RAs includes short-acting GLP-1 analogs, such as exenatid, and long-acting GLP-1 analogs, such as abirutide and liraglutide. There are several main mechanisms of glucose control. For instance, one mechanism involves promoting the production and secretion of GLP-1RAs, which significantly enhances glucose-induced insulin secretion by their interaction with specific receptors on pancreatic beta cells, which then simultaneously activates sensitive nerves and glucose receptors on beta cells to exert hypoglycemic effects (Ahrén 2004). The effects of GLP-1RAs are concentration-dependent. GLP-1RAs do not induce insulin secretion when the blood glucose concentration is less than 3.6 mmol/L. Therefore, they do not cause severe hypoglycemia. Another mechanism is the inhibition of glucagon secretion, where GLP-1RAs act on islet α cells and inhibit the release of glucagon (Ahrén 2004). They also act on pancreatic islet δ cells to promote the secretion of somatostatin, which can act as parastrin to inhibit glucagon. The third mechanism entails the protection of islet β cells through the stimulation of their proliferation and differentiation as well as the promotion of their regeneration and repair (Flamez et al., 1998). β cells are protected by maintaining β cell morphology and inhibiting the expression of pre-apoptotic genes. The fourth mechanism is activated when GLP-1RAs reduce the concentration of free fatty acids and liver glucose production, thereby reducing insulin resistance (Mette et al., 2002). The final mechanism is slowing gastric emptying, inhibiting the inappropriate release of postprandial glucagon, and possibly inhibiting the appetite control center of the brain (Schirra et al., 2006) CLP-1 analogs are currently used for patients with type 2 diabetes (T2DM), as well as for patients with obesity. In 2018, the American Diabetes Association/European Association for the Study of Diabetes new consensus recommended GLP-1RAs as the first choice for glucose-lowering therapy in T2DM patients with ASCVD (Davies et al., 2018).

In the event of acute infection, the pro-inflammatory factor IL-6 can induce the secretion of GLP-1 to optimize the level of blood sugar (Kahles et al., 2014). In a mouse model of acute kidney injury caused by sepsis, the expression of GLP-1R in the renal tubules increases in the early stage of sepsis, and the endogenous GLP-1 regulation reduces kidney injury (Choi et al., 2019). In addition, dipeptidyl peptidase 4 (DPP-4), a drug that inhibits the degradation of GLP-1, reduces kidney damage through antiapoptotic, immunological, and antioxidative changes (Glorie et al., 2012). After LPS-induced sepsis in rats with *DPP-4* knockout, the administration of GLP-1 RAs can increase cardiac cAMP levels, improve cardiovascular function in animals with endotoxemia, and improve the prognosis of endotoxemia (Ku et al., 2010).

## Effect of GLP-1RAs on Blood Glucose in Patients With Sepsis

**FIGURE 1:**
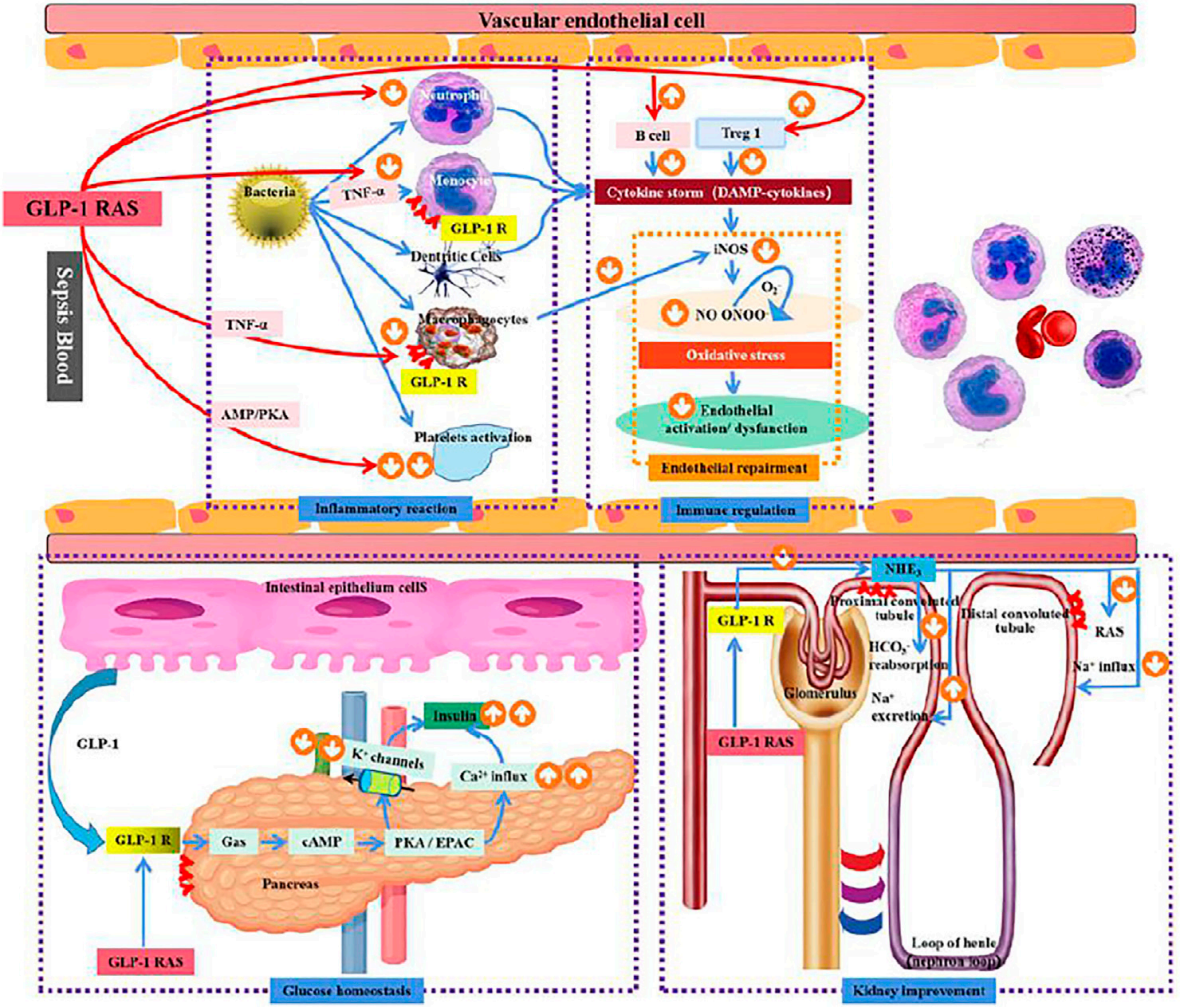
Mechanism of GLP-1R in sepsis.

L-cells that secrete GLP-1 are a type of multifunctional endocrine cells that only exist in the intestinal mucosal epithelium. GLP-1 is mainly secreted upon stimulation by intestinal nutrients, but recent studies have found that inflammation can also stimulate the secretion of incretin (Kahles et al., 2014) (Figure 1). As the only carrier of L-cells, the intestine is one of the most important targets of organ function in sepsis. The gastrointestinal tract of patients with sepsis is often the first organ affected, which often manifests as gastrointestinal intolerance. Patients in the acute stage of the disease are often in a state of fasting due to unstable hemodynamics, and the decreased secretion of endogenous intestinal glucagon hormone is an important factor leading to the fluctuation of blood glucose levels in patients with sepsis. GLP-1 expression in patients in the early stage of sepsis can quickly increase to more than twice that in healthy people, reaching as high as 9.5 times higher in patients with severe sepsis. This may be a feedback protection mechanism when the body suffers severe damage (Perl et al., 2018). To resist the inflammatory reaction, as the disease progresses, the GLP-1 system is inhibited, causing hyperglycemia. Most patients with sepsis have stress hyperglycemia, which reduces the ability to resist pathogen invasion (Jafar et al., 2016). Acute hypoglycemia increases oxidative stress, platelet aggregation, production of pro-inflammatory cytokines, and the expression of vascular adhesion molecules (Gogitidze Joy et al., 2010; Wright et al., 2010). If blood glucose is unstable in patients with sepsis, nosocomial mortality is remarkably increased (Leonidou et al., 2007). Furthermore, in patients with sepsis, increased glucagon levels caused by a decreased endogenous secretion of intestinal glucagon have been associated with disease severity and poor prognosis (Jung and Park. 2015; Perl et al., 2018). In animal models of sepsis, intestinal glucose can induce the decrease of intestinal glucose levels and blood sugar, and GIP promotes insulin secretion, reduces glucagon secretion, increases insulin secretion, weakens the systemic inflammatory response, and improves hemodynamics (Shah et al., 2017). Results of previous studies suggest that sepsis at a steady state depends on endogenous glucose and blood sugar levels, as well as hormonal regulation. Patients with stress hyperglycemia caused by sepsis may benefit from the exogenous supplementation of GLP-1RAs to optimize blood glucose control while reducing blood glucose variation rate, and the protective effect of GLP-1RAs on multiple organ functions may improve the prognosis of sepsis.

The effect of GLP-1Ras on sepsis: 1) The effect of blood glucose homeostasis: The endogenous GLP-1 and exogenous GLP-1Ras secreted in the small intestine bind to GLP-1R on the surface of the pancreas, stimulate Ca2+ influx and reduce K+ outflow through cAMP activation of PKA/EPAC signaling pathway, promote insulin secretion, and regulate blood glucose homeostasis 2) Inflammation response: GLP-1R is expressed in macrophages and monocytes, and can inhibit the release of inflammatory factors through TNF-α, AMP/PKA and other pathways to reduce systemic inflammatory response. Meanwhile, macrophages can inhibit the release of iNOS to improve vascular endothelial cell dysfunction. GLP-1 RAS can also reduce platelet activation through the AMP/PKA pathway and reduce microthrombosis in sepsis patients. 3) Immune function: GLP-1R is expressed in B lymphocytes and T lymphocytes. The activation of GLP-1R can promote the proliferation of B lymphocytes and T lymphocytes, especially the expression of Treg1, to inhibit systemic inflammatory response in sepsis patients. 4) Effects of GLP-IR activation on endothelial cells: GLP-IR activation can reduce oxidative stress of endothelial cells by reducing iNOS secretion, thereby improving endothelial cell dysfunction; 5) Renal effects: the expression of GLP-1 receptor in renal tubules, GLP-1 RAS can prevent inflammation and septicaemic induced AKI by blocking the sodium-hydrogen exchanger (NHE3), enhance the excretion of sodium in renal tubules, and reduce RAS activation; Thus play the role of kidney protection.

## Effect of GLP-1RAs on Organ Function

GLP-1RAs are relatively safe for controlling blood glucose. Current studies suggest that the GLP-1R is distributed in all organs, mainly located on the β cells in the pancreas. In kidneys and lungs, GLP-1R is expressed in the smooth muscle cells of the arteries and arterioles. In the heart, GLP-1R is located on the muscle cells of the sinoatrial node. In the gastrointestinal tract, the expression of GLP-1R in Bruner's gland of the duodenum has been detected (pyke et al., 2014). A large number of studies have shown that GLP-1RAs have protective effects on multiple organs. In one study of patients with cardiovascular disease, treatment with a GLP-1 analog (liraglutid) resulted in significantly lower rates of non-fatal myocardial infarction, non-fatal stroke, and hospitalization for heart failure, compared with the placebo groups (Marso et al., 2016). Many GLP-1 analogs have been shown to reduce the incidence and progression of diabetic nephropathy compared with the results obtained with the conventional therapy for patients with T2DM(Orsted et al., 2017). In a mouse model of ischemic stroke, GLP-1RA treatment was shown to exert neuroprotective effects and prevent memory impairment (Teramoto et al., 2011). Since GLP-1 receptors are distributed in the pancreas, kidneys, lungs, heart, gastrointestinal tract, and other organs (Pyke et al., 2014), an increasing number of studies have focused on the effect of GLP-1RAs on organ function, and the results have confirmed the protective effect of GLP-1RAs on the heart (Marso et al., 2016), kidneys (Orsted et al., 2017) and the nervous system (Teramoto et al., 2011). In comatose out-of-hospital cardiac arrest patients with elevated blood glucose levels, GLP-1 analogs can reduce blood glucose and improve lactic acid clearance, possibly improving neurological prognosis through its effect on glycolysis and hemodynamics (Wiberg et al., 2017). In animal experiments, GLP-1 has been shown to improve the metabolic efficiency of myocardial glucose, reduce systemic and pulmonary vascular resistance, and activate the ischemic preconditioning pathway (Aravindhan et al., 2015) GLP-1-mediated positive effects on cardiac function have been observed in clinical studies, and the application of a GLP-1 analog in patients with ischemic injury resulted in improved left ventricular function and reduced infarct size (Lazaros. 2004). In the LEADER trial, patients with diabetes treated with liraglutide had a lower incidence of major adverse cardiovascular events, including cardiovascular death, compared to the placebo group (Marso et al., 2016). With the increasing understanding of the roles and functions of GLP-1RAs, some scientists have begun to pay attention to the effect of GLP-1RAs on the organs in the pathological process of sepsis. GLP-1, a marker of early intestinal mucosal injury, was significantly increased after LPS injection in a mouse model of intestinal injury induced by lipopolysaccharide (IPOLPS) (Lebrun et al., 2017). There are a large number of bacteria in the intestinal tract, and when the intestinal mucosal barrier is damaged, LPS produced by bacteria can enter the blood circulation, leading to an increase in GLP-1 levels, which can improve mucosal integrity and reduce local and systemic inflammation (Lebrun et al., 2017). Exogenous GLP-1 can protect the intestinal tract from oxidative damage, regulate intestinal homeostasis through local effects, and restore intestinal integrity (Deniz et al., 2015). In a rat model of acute kidney injury in early sepsis, Glp-1 receptor expression in renal tubules increased, and the induction of Glp-1 receptor expression prevented inflammation and sepsis-induced AKI (Choi et al., 2019), a Glp-1 analog, by blocking sodium hydrogen exchanger, enhancing renal tubular sodium (Girardi et al., 2008), and reducing the activity of angiotensin (Jeppe et al., 2013); thus, playing a role in kidney protection. Also, the systemic inflammatory reaction caused by sepsis may lead to multiple organ function impairment in patients. Whether GLP-1RAs can benefit such patients warrants further clinical confirmation.

## Effect of GLP-1RAs on Inflammatory Response Induced by Sepsis

In an observational study of patients with sepsis, it was found that endogenous GLP-1 was activated during sepsis, and GLP-1 levels were significantly elevated in patients with sepsis who died early (2–4 days post-admission) (Perl et al., 2018). Scott Brakenridge et al. found that increased GLP-1 levels within 24 h of sepsis are closely associated with early death, and in survivors, a sustained increase in GLP-1 levels after 14 days was also associated with severe dysfunction after 6 months, and the changes were associated with IL-6 secretion (Brakenridge et al., 2019). GLP-1RAs inhibit TNFα-induced activation of NF-κB and decrease the expression of adhesion molecules, including VCAM-1, ICAM-1, and E-selectin (Hattori et al., 2010). In animal models and *in vitro* experiments of sepsis, GLP-1 analogs inhibited TNFα and LPS-induced monocyte adhesion, thereby reducing vascular endothelial injury (Krasner et al., 2014). Inhibition of the NF-κB pathway and the secretion of inflammatory cytokines in macrophages improve inflammatory macrophage-derived insulin resistance (Guo et al., 2016), while the inhibition of LPS-induced inflammatory pathways and iNOS expression, and the decrease in AMPK activation improve vascular dysfunction and alleviate oxidative stress in endotoxemia rats (Steven et al., 2015). The GLP-1 receptor is expressed on eosinophils and neutrophils, and GLP-1RAs can reduce the expression of eosinophil surface activation markers after LPS stimulation, and reduce the production of IL-4, IL-8, and IL-13, thereby reducing the inflammatory response (Mitchell et al., 2017). Activation of the GLP-1 receptor reduces the expression of LPS-induced pro-inflammatory cytokines, including IL-1β, IL-6, TNF-α, and IFN-γ, *in vivo* and *in vitro* (Ofer et al., 2015; Young-Sun and Hee-Sook 2016). GLP-1RA activates the GLP-1 receptor in platelets, through an cyclic adenosine monophosphate/protein kinase (AMP/PKA)-dependent mechanism to prevent systemic inflammation, vascular dysfunction, and end-organ damage, thereby significantly reducing the microvascular thrombosis induced by endotoxin blood disease and mortality (Steven et al., 2016) (Tests on human vascular endothelial cells exposed to LPS have demonstrated that GLP-1 analogs reduce vascular permeability and lower the levels of pro-inflammatory cytokines (Wonhwa et al., 2016).

## Effect of GLP-1RAs on Immune Response Induced by Sepsis

GLP-1R is expressed in macrophages, monocytes, B cells, and T cell lymphocytes. The lack of GLP-1R signal will impair the ability of mouse lymphocytes to divide and proliferate. GLP-1R activation causes increased cAMP levels, which activates and regulates Treg function and promotes peripheral T cell proliferation (Hadjiyanni et al., 2010). Treg cells have obvious anti-inflammatory effects, and can secrete anti-inflammatory cytokines, such as IL-4, IL-10, and TGF-β, to inhibit auto-inflammatory response and prevent pathological immune responses that cause tissue destruction. (Sakaguchi 2004). After activation, GLP-1R can inhibit the release of pro-inflammatory cytokines IL-2, IL-17A, interferon gamma, and tumor necrosis factor alpha triggered by anti-CD3 and -CD28 antibodies produced by intestinal intraepithelial lymphocytes (Yusta et al., 2015). The results of studies on sepsis-related cells and animal models suggested that GLP-1RAs activate the cAMP/PKA pathway to reduce the expression of iNOS in Raw264.7 macrophages induced by LPS (Chang et al., 2013). GLP-1RAs inhibit the activation of leukocytes and the differentiation of antigen-presenting cells, thereby reducing the adhesion and infiltration of activated leukocytes on the vascular endothelium, and reducing endothelial cell damage (Steven et al., 2015). GLP-1 RAs inhibit platelet activation through the cAMP/PKA pathway, reduce endotoxemia-induced microvascular thrombosis, and reduce the occurrence of diffuse intravascular coagulation, thereby preventing systemic inflammation, improving vascular dysfunction, reducing organ injure, and improving the outcome of patients with sepsis (Steven et al., 2017). GLP-1 receptor is also expressed in CD14-positive monocytes of healthy people (Arakawa et al., 2010). In endotoxemia, after LPS binds to CD14 on the surface of monocytes and phagocytoses, the TLR-4/CD14 complex is cleaved into sCD14 (the water-soluble form of CD14). The increase in the level of circulating sCD14 is associated with the high fatality rate of septic shock caused by Gram-negative bacteria (Landmann et al., 1995). In sepsis caused by Gram-negative bacteria, it was found that the GLP-1 system is over-activated. On the one hand, its over-activity is related to increased sCD14 levels, which activates the innate immune response to resist the invasion of pathogenic microorganisms. On the other hand, it may reduce excessive inflammation to prevent immune system disorders and the adverse consequences of sepsis (Bloch et al., 2021).

Due to the complex etiology of sepsis, the need to balance infection and inflammation in the treatment process, and the abnormal fluctuation of blood glucose due to endocrine abnormalities during the course of the disease, maintaining “reasonable” blood glucose in sepsis patients is still a challenge. Current studies suggest that hyperglycemia is associated with a variety of poor clinical outcomes, and critically ill patients with hyperglycemia have a higher mortality rate than patients with normal blood glucose (Krinsley. 2003). However, the incidence of hypoglycemia is also significantly increased in patients using insulin, and previous studies have shown that hypoglycemia is an independent predictor of hospitalization and one-year mortality in critically ill patients (Krinsley et al., 2007; Waeschle et al., 2008; Park et al., 2012). Selection in critically ill patients can effectively control blood glucose without increasing the risk of hypoglycemia, which is of great significance for improving the prognosis of patients with sepsis. GLP-1 analogs can regulate immune response and the release of inflammatory factors in severely ill patients, especially in patients with sepsis, and exert organ protection effects, which may benefit patients. However, there is still a lack of large-scale high-quality clinical studies, and ascertaining the clinical safety and effectiveness of GLP-1RA treatment warrants further study.
